# Refractory Primary Duodenal Plasmacytoma Displaying t(11;14): A Report of a Rare Case

**DOI:** 10.7759/cureus.84443

**Published:** 2025-05-20

**Authors:** Nora El Maachi, Marlene Ochmann, Claire Belchet

**Affiliations:** 1 Hematology, Mohammed V Military Hospital, Rabat, MAR; 2 Hematology, Centre Hospitalier Régional (CHR) d'Orléans, Orléans, FRA; 3 Pathology and Laboratory Medicine, Centre Hospitalier Régional (CHR) d'Orléans, Orléans, FRA

**Keywords:** duodenal, plasmacytoma, primary, refractory, t(11:14)

## Abstract

Extramedullary plasmacytoma (EMP) involving the digestive tract is a very rare entity that represents only a very small fraction of multiple myeloma (MM) cases. We describe an unusual case of a 71-year-old man with EMP involving the duodenum who initially presented to the emergency room with melena. The final diagnosis was duodenal plasmacytoma with t(11;14), and the diagnosis was reached by fibroscopy with biopsy and immunohistochemistry (IHC). The patient received local radiotherapy and showed failure three months after treatment. Then, he was administered daratumumab-based treatment associated with Revlimid and dexamethasone (DRD).

To our knowledge, this is the first report showing duodenal involvement by EMP with t(11;14).

## Introduction

Extramedullary plasmacytoma (EMP) is a rare type of plasma cell neoplasm that arises in soft tissues without the involvement of the bone marrow [1]. Gastrointestinal (GI) tract involvement in EMP is particularly uncommon, accounting for less than 1% of multiple myeloma (MM) cases [2]. EMP is known to be highly sensitive to radiotherapy [1]. Due to its rarity, we present a case involving a 71-year-old man diagnosed with duodenal plasmacytoma carrying the t(11;14) translocation. Initially, the patient was treated with localized radiotherapy, which did not yield a response. Subsequently, he was administered a daratumumab-based treatment associated with Revlimid and dexamethasone (DRD).

## Case presentation

**Figure 1:**
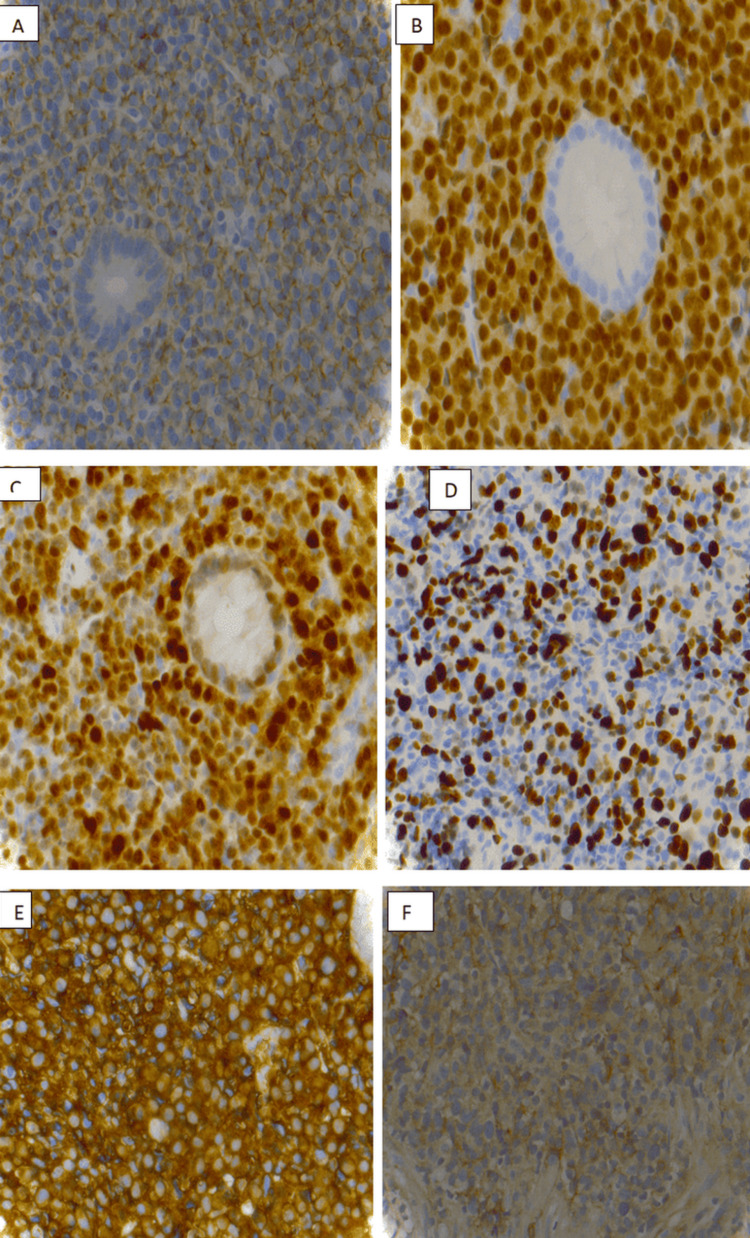
(A) Positive immunostaining for the plasma cell marker CD138 (400×). (B) Positive plasma cell marker MUM1 (400×). (C) Positive plasma cell marker cyclin D1 (400×). (D) Plasma cell marker Ki67 (400×). (E) Fine membrane granular positivity for kappa immunoglobulin light chain (400×). (F) Fine membrane granular positivity for lambda CD138, cluster of differentiation 138; MUM1, multiple myeloma oncogene-1

A 71-year-old man with a medical history of hypertension, multinodular goiter, chronic bronchitis, polycystic kidney disease, and bilateral orchiepididymitis presented to the emergency room with melena and hematochezia. He did not report any history of bleeding, abdominal operation, or use of antithrombotic agents or nonsteroidal anti-inflammatory drugs (NSAIDs). On physical examination, the patient appeared pale, although hemodynamic status remained stable. Mild tenderness was noted around the epigastric region. Laboratory tests revealed severe normocytic normochromic anemia with a hemoglobin level of 6.4 g/dL. An upper GI endoscopy showed a hemi-circumferential duodenal ulcer (Forrest classification Ib) without *Helicobacter pylori* infection. No hemostatic intervention was possible. Multiple endoscopic biopsy samples were taken, and histology showed a chorion massively infiltrated by a small lymphoid population, with diffuse architecture and strong plasma cell differentiation without lymphoepithelial lesion. Immunohistochemistry (IHC) was negative for cluster of differentiation 20 (CD20), CD79a, CD5, CD56, CD23, and CD10 and B-cell lymphoma 6 (BCL-6)-negative for B-cell markers. Immunostaining was strongly positive for the plasma cell marker CD138 with monotype immunoglobulin A (IgA) kappa consistent with plasmacytoma, multiple myeloma oncogene-1 (MUM1) (100%), and cyclin D1 (100%) (Figure 1). Anti-CD117 labels scattered mast cells in the chorion. A test for Epstein-Barr virus (EBV) was negative.

Fluorescence in situ hybridization (FISH) for multiple myeloma markers revealed a positive result for translocation t(11;14), confirming the presence of translocation *CCND1-IGH*. Bone marrow aspirate showed 2% poorly differentiated plasma cells. Cytofluorometry was without atypia. Serum protein electrophoresis showed no monoclonal protein.

Serum free light chain analysis showed elevated kappa (34 mg/L) and lambda (20 mg/L) levels, with a mildly increased kappa/lambda ratio. Routine laboratory tests, including serum lactate dehydrogenase (LDH), beta-2 microglobulin, serum calcium, and renal function, were all within normal limits (Table 1).

**Table 1:** Routine laboratory tests eGFR: estimated glomerular filtration rate

Routine laboratory tests	Values
Lactate dehydrogenase	135 (U/L)
Beta-2 microglobulin	3 mg/L
Calcium	8.5 mg/dL
Renal function	eGFR: 90

**Figure 2:**
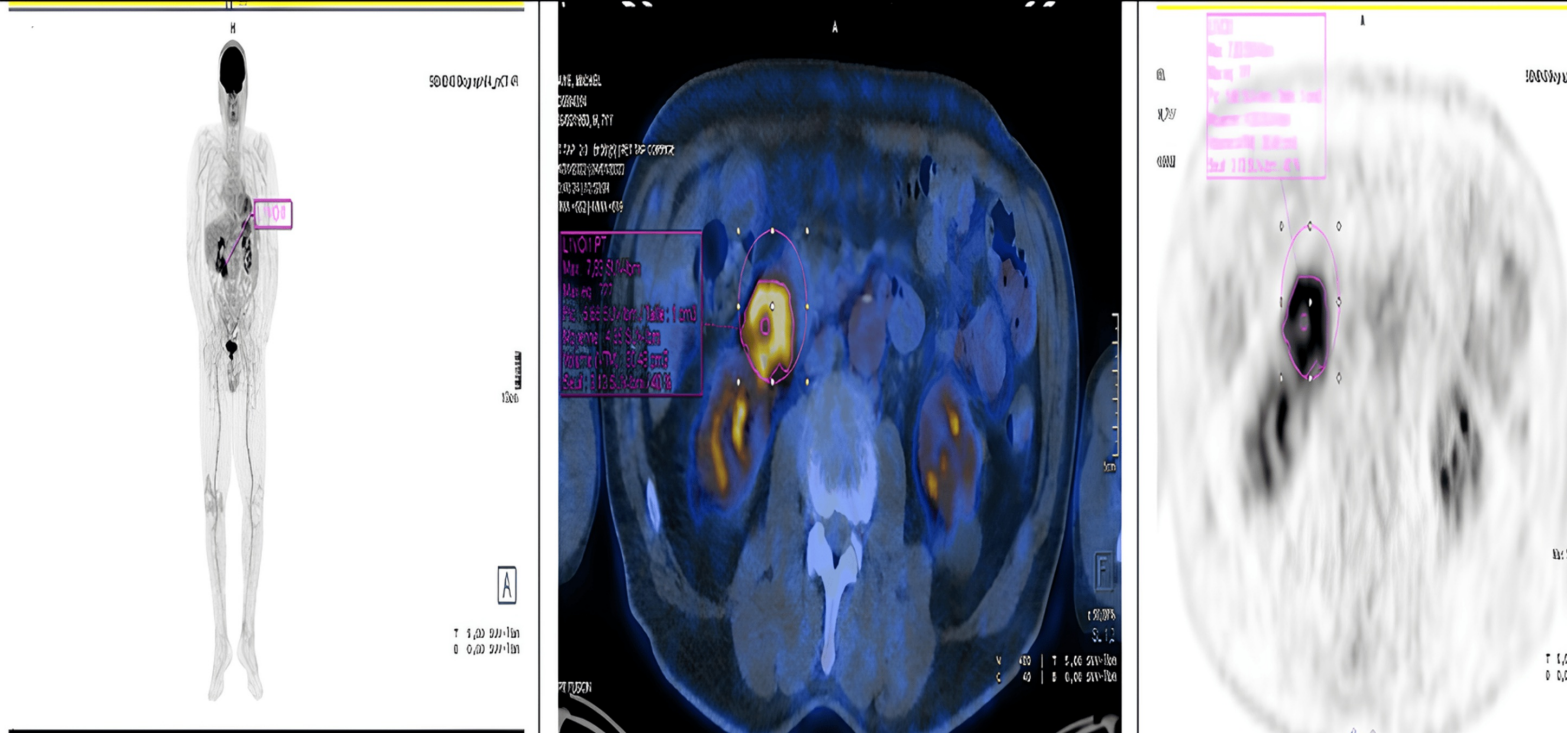
PET/CT at diagnosis showed pathological hypermetabolism at the height of the tumoral infiltration of the second duodenum (SUVmax: 16.8) PET/CT, positron emission tomography/computed tomography; SUVmax, maximum standardized uptake value

Positron emission tomography/computed tomography (PET/CT) revealed pathological hypermetabolism corresponding to the tumoral infiltration in the second portion of the duodenum, with a maximum standardized uptake value (SUVmax) of 16.8. Additionally, a hypermetabolic focus was noted in the right prostatic lobe, warranting further evaluation with total prostate-specific antigen (PSA) testing. No other suspicious metabolic abnormalities were identified on whole-body imaging (Figure 2).

The patient underwent intensity-modulated radiation therapy (IMRT) with a total dose of 50 Gy, delivered in five fractions over five weeks, beginning in March 2022. As no *Helicobacter pylori* infection was identified, eradication therapy was not initiated.

**Figure 3:**
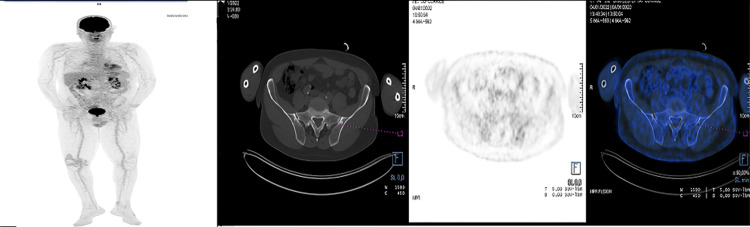
PET/CT three months after radiotherapy revealed a decrease in size and fixation of tumor hypermetabolic infiltration of the duodenum (SUVmax: 7.2 versus 16.8). Partial metabolic response to duodenal injury. The appearance of hypermetabolic bone lesions of the left iliac bone and the lower extremity of the left femur, without paramedullary extension PET/CT, positron emission tomography/computed tomography; SUVmax, maximum standardized uptake value

Follow-up PET/CT imaging in June 2022 demonstrated a decrease in both the size and metabolic activity of the duodenal lesion, with the SUVmax reduced from 16.8 to 7.2, indicating a partial metabolic response. However, new hypermetabolic bone lesions were observed in the left iliac bone and the distal femur, without evidence of paramedullary extension (Figure 3).

A follow-up upper gastrointestinal endoscopy in June revealed an ulcerated lesion with a fibrinous base originating at the genu superius, along with ulcerated and stenotic infiltrative involvement of the duodenal mucosa extending to the second portion (D2). Biopsies confirmed the persistence of the duodenal infiltration by monotypic kappa-restricted plasma cells expressing cyclin D1, consistent with the previously established diagnosis.

Repeat immunofixation electrophoresis for immunoglobulins (IgG, IgA, and IgM) and light chains (kappa and lambda) remained negative for monoclonal protein.

The patient was started on a DRD regimen comprising daratumumab 1800 mg subcutaneously on days 1, 8, and 15; lenalidomide (Revlimid) 25 mg orally on days 1-21; and dexamethasone 20 mg orally on days 1-2 and once weekly. Each treatment cycle was 28 days in duration. By the end of September, the patient had completed three treatment cycles without any reported toxicity. At the time of reporting, the patient remains under treatment, and no posttreatment evaluation data are yet available to assess therapeutic response.

## Discussion

Multiple myeloma (MM) with extramedullary plasmacytoma (EMP) is a rare clinical entity, accounting for approximately 3%-5% of all plasmacytomas [1,3]. EMP involving the gastrointestinal (GI) tract is particularly uncommon. Among reported cases, the small intestine is the most frequently affected site, followed by the stomach, colon, and esophagus [4].

Due to its rarity, there are currently no consensus guidelines for the management of intra-abdominal EMP [5]. Treatment strategies are typically guided by institutional protocols or individual clinician experience. Reported approaches include surgical resection, radiotherapy, systemic chemotherapy, or a combination of these modalities [6]. Optimal patient outcomes are best achieved through multidisciplinary team discussions involving surgery, hematology, oncology, pathology, and radiology.

To our knowledge, this is the first reported case of a duodenal plasmacytoma with a t(11;14) translocation. EMP is generally considered highly radiosensitive. Various radiotherapy protocols have been described, such as targeted radiotherapy delivering 40-50 Gy over four weeks, which was the approach used in our case [6,7]. However, our patient demonstrated a refractory response to radiotherapy, necessitating systemic treatment with a chemotherapy regimen.

This case also underscores the importance of a thorough diagnostic workup upon the identification of EMP. This should include serum and urine protein electrophoresis with immunofixation, free light chain analysis, bone marrow biopsy, and the radiographic evaluation of the axial skeleton to assess for systemic involvement and inform treatment planning.

Future directions

Further research is warranted to better understand the biological behavior of extramedullary plasmacytomas with t(11;14), as well as to evaluate the efficacy and safety of targeted therapies such as BCL-2 inhibitors in this context.

## Conclusions

Gastrointestinal involvement in EMP remains extremely rare. Given the potential sensitivity of t(11;14)-positive plasma cell neoplasms to BCL-2 inhibition, agents such as venetoclax may represent promising alternatives, particularly in refractory cases. Clinicians should consider the early integration of molecular diagnostics into treatment planning to identify high-risk features and guide individualized therapy.

Collaboration between international specialist centers is essential. Establishing registries and collecting standardized clinical data will improve future treatment strategies and improve outcomes for those with EMP.
